# Using the Electronic Health Record Patient Portal to Collect Advance Directives and Surrogate Specification

**DOI:** 10.1007/s11606-025-10165-w

**Published:** 2026-01-23

**Authors:** Annapoorna R. Chirra, Suzanne Manteuffel, Tracy Runnels, Leslie Sturgeon, Maria Caban Alizondo, Chi-Hong Tseng, Anne M. Walling, Neil S. Wenger

**Affiliations:** 1 Division of General Internal Medicine, University of California, Los Angeles, CA USA; 2 UCLA Health Information Services & Solutions, Los Angeles, CA USA; 3 Health Informatics & Information Management Services, UCLA Health Information Services & Solutions, Los Angeles, CA USA; 4 Division of General Internal Medicine and Health Services Research, University of California, Los Angeles, CA USA; 5 VA Greater Los Angeles Health System, Los Angeles, CA USA

**Keywords:** advance directive, patient portal, advance care planning, electronic health record

## Abstract

**Background:**

Advance directive completion and collection is a complex process including collecting documents, checking them for accuracy, and uploading them into a hospital or health system’s electronic health record (EHR). Hospitals and health systems need mechanisms to facilitate the process.

**Objective:**

To use the EHR patient portal and Health Informatics & Information Management System (HIIMS) personnel to collect and check the quality of advance care planning (ACP) documents and surrogate decision makers, and integrate them into the EHR.

**Design:**

Single arm time series quality improvement project.

**Intervention:**

Health IT, HIIMS, and the Advance Care Planning Program collaboratively developed a mechanism for ACP documents and surrogates submitted through the patient portal to be evaluated by HIIMS personnel who received training to process information entered by patients. HIIMS personnel integrate documents and surrogate information into the EHR or return a rejection message to the patient.

**Main Measures:**

Number of documents submitted, accepted, rejected, and accepted after resubmission, and the number of surrogates entered, updated, and removed.

**Key Results:**

Over 41 months from May 2021 to September 2024, 7274 ACP documents were uploaded via the patient portal (mean 177 documents per month): 4700 (65%) accepted on first submission and 2574 (35%) rejected. Of rejected documents, 397 (15%) were resubmitted and accepted within the month. Over time, the proportion of accepted documents increased. During the study period, 12,509 surrogates were added (mean 305 per month), 1790 were updated, and 911 were removed.

**Conclusion:**

Engaging HIIMS personnel can facilitate ACP document capture through a common EHR portal, including quality checks and patient feedback.

**Supplementary Information:**

The online version contains supplementary material available at 10.1007/s11606-025-10165-w.

## INTRODUCTION

In order to guide care for patients, and particularly to involve them in deciding what treatments they want when seriously ill and toward the end of life, advance care planning—the process of understanding and sharing personal values, life goals, and preferences regarding future medical care that, if appropriate, yields written documents and medical orders to guide care—is recommended for adult patients including completion of an advance directive.^1^ Advance Care Planning has been associated with enhanced communication, documentation of goals that guide care,^2^ increased concordance between patients’ preferences and end-of-life care received,^3^ and better bereavement adjustment.^4^ Completion of the advance directive is an important component of advance care planning because it makes the specification of the health care agent official and its formulaic components serve to indicate the importance of the process. In addition, other documents such as Physician Orders for Life Sustaining Treatment (POLST), which contains medical orders to be applied in an emergency, and some conservatorships also direct treatment decisions and these need to be available at the time and location of care.

However, the many steps in advance directive completion—seriously considering the end of life with a pen in hand, choosing a health care agent, signing the document, and having it signed by witnesses or a notary—have long been known to pose obstacles.^5^ Because the advance directive requires the capable patient’s signature and those of witnesses or a notary, it is not well-suited to on-line completion. This creates complexity in collecting documents, checking them for accurate completion, and uploading them into the proper spot in a hospital or health system’s electronic health record (EHR). Failing these steps, advance directive documents will not contribute to care when and where needed because they are unavailable^6^ or incomplete. Furthermore, difficulty in advance directive completion leaves information about preferred decision makers in an “unofficial” form until it is recorded in a formally completed and valid advance directive. To promote completion and collection of advance care planning documents, hospitals and health systems need mechanisms to facilitate the process.^7^

UCLA Health built on the infrastructure in Epic, an EHR used commonly by large health systems, to construct a mechanism to collect advance care planning information and documents in the context of the clinical workflow and also to encourage patients to submit surrogate information and documentation. The build incorporated Health Informatics & Information Management Services (HIIMS) personnel in the collection and quality assurance of advance care planning documents. We describe this advance care planning support system and the effect on advance care planning documentation in the health system.

## METHODS

UC Health is an academic medical center with five hospitals, more than 280 outpatient clinics, and annually more than 4 million outpatient visits and 40,000 admissions. We introduce the health system advance care planning infrastructure in Epic and then the mechanism developed to accept patient input in the MyChart patient portal of advance care planning documents and surrogate specification. The study was deemed not human research by the UCLA Institutional Review Board (25–0189).

### Advance Care Planning Infrastructure

We modified the Epic Advance Care Planning activity in the EHR to distinguish between a Health Care Agent who is officially named in a valid advance directive and a surrogate named by a patient who has not yet been captured in a valid advance directive (defined by the health system as a “Designated Surrogate” (Box 1)). The latter is commonly elicited during a clinical encounter in which a clinician initiates an advance care planning discussion or while discussing prognosis. Terms and definitions for these classifications of surrogate decision maker were embedded in the Advance Care Planning activity along with a “Default Decision Maker” for a patient with permanent incapacity who had never specified to whom to turn (Appendix Figure 1). The Advance Care Planning activity also includes all advance care planning documents and Goals of care notes. These are also shown on a tab in Chart Review, which preceded the Epic Advance Care Planning activity by 5 years at UCLA Health, for ease of access during clinical care. The inclusion of an advance directive or POLST in the Advance Care Planning activity is illuminated on the Storyboard (the first page of the EHR). 

**Table:** Box 1 Designated Surrogate

The Designated Surrogate is different than the Health Care Surrogate, which according to California law can be verbally communicated to the supervising attending physician for an “episode of care” that is widely interpreted as a hospitalization. The supervising attending physician must document that in the medical record. A mechanism for such documentation has been built into the UCLA Health EHR, but is not described in this report	

The “Advance Directive” page in the MyChart patient portal, which includes an introduction to advance care planning and advance directives as well as additional information and a link to more detailed sources, includes a mechanism to upload a completed advance directive or POLST. There also is a mechanism for a patient to write in their surrogate decision maker (should they become incapable of decision making) and to choose an appropriate label (Health Care Agent or Designated Surrogate) for that individual. Patients also can remove surrogate decision makers. The page included information to guide the patient in these activities (Appendix Figure 2).

The MyChart Advance Directive page invited patients using the portal to submit documents and surrogate names, but no special notification was provided to patients concerning the HIIMS infrastructure for processing the documents and information. Primary care providers were made aware of this new infrastructure and were asked to prompt their patients to submit advance directives through the patient portal.

### HIIMS Infrastructure for Processing Advance Care Planning Documents and Surrogate Information

In collaboration, Health IT, HIIMS and the Advance Care Planning Program developed a health system-wide mechanism for advance care planning documents and surrogate information submitted through the patient portal to be evaluated by HIIMS personnel and integrated into the EHR or rejected and a message returned to the patient. HIIMS personnel receive data integrity training; some have certification as Registered Health Information Administrators. These individuals monitor the quality of documents and ensure proper placement in the EHR. The submitted advance care planning document is transmitted to a HIIMS InBasket and these documents are reviewed by HIIMS personnel for validity: the advance directive document is complete, signed and dated by the patient, and witnessed or notarized. The valid advance directive is uploaded into the appropriate area in the Advance Care Planning activity, the Chart Review tab and so it is viewable to the patient on MyChart. The HIIMS staff enters the Health Care Agent into the Advance Care Planning activity. The label is changed to Health Care Agent if this individual had been listed as the Designated Surrogate. A valid POLST (Section A concerning resuscitation filled in; signed by the physician, nurse practitioner or physician’s assistant; and signed and dated by the patient or appropriate surrogate) is uploaded by HIIMS into the appropriate area in the Advance Care Planning activity, the Chart Review tab and so it is viewable to the patient on MyChart. If an advance directive or a POLST is found to be invalid (or if a different sort of document is uploaded and mislabeled) then the document is rejected, and a message is sent to the patient. Decision rules for handling advance directive and POLST (and other documents) are detailed in Table 1.

**Table 1:** HIIMS Assessment and Handling of ACP Documents

**Documents uploaded**	**HIIMS action**	**Accept: document type to assign**	**Deny: reason**	**Deny: reason given to patient**
**Advance directive**				
Advance directive complete (CA or another state)	Accept	Advance Directive Enduring		
Advance directive signed (CA or another state)	Deny		Missing Signature	Adv directive not signed or dated. Please correct and re-submit document
Advance directive undated (CA or another state)	Deny		Missing Date	Adv directive not signed or dated. Please correct and re-submit document
Advance directive without notary or two witnesses (CA or another state)	Deny		Missing Notary or Witness	Adv directive without notary or witnesses. Please correct and re-submit document
Advance directive blank	Deny		Missing Documentation	Adv directive without content. Please correct and re-submit document
**Power of attorney**				
Power of attorney for health care complete	Accept	Advance Directive Enduring		
Power of attorney for health care unsigned (CA or another state)	Deny		Missing Signature	Adv directive not signed or dated. Please correct and re-submit document
Power of attorney for health care undated (CA or another state)	Deny		Missing Date	Adv directive not signed or dated. Please correct and re-submit document
Power of attorney for health care without notary or two witnesses (CA or another state)	Deny		Missing Notary or Witness	Adv directive without notary or witnesses. Please correct and re-submit document
Power of attorney for health care blank	Deny		Missing Documentation	Adv directive without content. Please correct and re-submit document
**Living will**				
Living will care complete	Accept	Advance Directive Enduring		
Living will unsigned or undated	Deny		Missing Date	Adv directive not signed or dated. Please correct and re-submit document
Living will for blank	Deny		Missing Documentation	Adv directive without content. Please correct and re-submit document
**POLST**				
POLST complete	Accept	POLST		
POLST No Section A	Deny		Missing Documentation	POLST incomplete. Please contact your physician to complete the POLST
POLST missing patient/surrogate or MD/NP/PA signature	Deny		Missing Signature	POLST not signed or dated. Please contact your physician to complete the POLST
POLST missing patient/surrogate or MD/NP/PA dates	Deny		Missing Date	POLST not signed or dated. Please contact your physician to complete the POLST
**Conservatorship**				
Conservatorship	Accept	Conservatorship		
**Other document**				
Financial power of attorney	Deny		Invalid Form	Not advance directive document
Other Documents	Deny		Invalid Form	Not advance directive document

The table shows HIIMS decision rules for uploaded documents, label for accepted documents, and reasons and responses to patient for rejected documents

*HIIMS*, Health Informatics & Information Management System; *POLST*, Physician Orders for Life Sustaining Treatment

HIIMS personnel also process the surrogate identification information entered by the patient into MyChart. Patients can enter a Health Care Agent (and first and second alternates) or a Designated Surrogate along with contact information for this individual. HIIMS staff justify this information with the advance directive (if any) in the EHR. If the patient enters an individual that they label as a Health Care Agent but there is no advance directive in the EHR (and an advance directive is not uploaded at the same time), then this individual will be labeled as a Designated Surrogate in the Advance Care Planning activity and this label will be visible on MyChart. If the patient submits the name of a Health Care Agent that conflicts with the advance directive in the EHR, this information will not lead to a change in the Advance Care Planning activity and the patient will receive a message stating “Health Care Agent entered conflicts with advance directive in the UCLA record. Please submit an advance directive designating this Health Care Agent.” Decision rules for handling surrogate specification are detailed in Table 2.

**Table 2:** HIIMS Assessment and Handling of Surrogates Specified by Patients

Surrogate specification	HIIMS action	Edit & Accept: relationship to assign	Edit & Accept: ACP activity action	Deny: reason	Deny: reason given to patient	
**Health Care Agent**						
Matches advance directive document	Accept					
Matches advance directive document but a different person is listed as Health Care Agent or Designated Surrogate	Edit & Accept	Health Care Agent	Delete relationship on the person not in the advance directive			
No advance directive	Edit & Accept	Designated Surrogate	Update relationship to designated surrogate			
Conflicts with advance directive	Deny			Relationship not supported by current documentation	Health Care Agent entered conflicts with advance directive in the UCLA record. Please submit an advance directive designating this Health Care Agent	
**Designated Surrogate**						
No advance directive	Accept					
Matches Health Care Agent in advance directive	Edit & Accept	Health Care Agent	Update relationship to Health Care Agent			
Conflicts with advance directive Health Care Agent	Deny			Relationship not supported by current documentation	Designated Surrogate entered conflicts with the Health Care Agent designated in the advance directive in the UCLA record. Please submit an advance directive designating this individual or contact your physician	
Conflicts with a different person listed as Health Care Agent or Designated Surrogate	Deny			Relationship not supported by current documentation	Designated Surrogate entered conflicts with the Health Care Agent or Designated Surrogate listed in the UCLA record. Please contact your physician	
Surrogate specification	InBasket action		Edit & Accept: relationship to assign	Edit & Accept: ACP activity action	Deny: reason	Deny: reason given to patient
**Default Surrogate**						
Matches Health Care Agent in advance directive	Edit & Accept		Health Care Agent	Update relationship to Health Care Agent		
No advance directive	Deny				Relationship not supported by current documentation	Default Surrogate is not a category to be selected by patient. Please select a different designation or consult your physician
Conflicts with advance directive	Deny				Relationship not supported by current documentation	Default Surrogate is not a category to be selected by patient and your designation conflicts with the Health Care Agent designated in the advance directive in the UCLA record. Please consult with your physician
Conflicts with a different person listed as Health Care Agent or Designated Surrogate	Deny				Relationship not supported by current documentation	Default Surrogate is not a category to be selected by patient and your entry conflicts with the Health Care Agent or Designated Surrogate listed in the UCLA record. Please contact your physician

The table shows HIIMS decision rules for specified surrogates, label for accepted surrogates, and reasons and responses to patient for rejected surrogates

*HIIMS*, Health Informatics & Information Management System; *POLST*, Physician Orders for Life Sustaining Treatment

Educational materials were developed for HIIMS personnel, and training sessions were conducted that included an introduction to advance care planning and advance directives, and the HIIMS tasks concerning submitted documents and surrogate information. Discussion included nuances of what constituted a valid advance care planning document, whether newly submitted documents and surrogates overrode or were supplemental to information in the EHR, and document dates and timing of submission. HIIMS personnel developed and modified responses to patients for documents and surrogate names that were rejected to facilitate understanding and resubmission. A living FAQ document (Appendix Table 1) was developed by HIIMS personnel, and follow-up sessions were conducted to discuss unusual cases. A Job Aid reflecting the elements of training and standardized rules is available from the authors.

### Analysis

We produced run charts to evaluate the number of advance directive and POLST forms received, accepted, and rejected per month. We also tabulated the number of rejected documents that were resubmitted and accepted within the calendar month. We also evaluated the number of surrogates submitted that were recorded in the EHR, the number of surrogates rejected, and the number of surrogates removed by the patient. These span May 2021, the month that the build went live, through September 2024. All patients with MyChart portal access were included in the analysis. Linear regression analysis was used to estimate the average change per month in the number of advance care planning documents submitted, the number of surrogate specifications submitted, the proportion of submitted advance care planning documents accepted (either on first submission or after resubmission), and the proportion of rejected advance care planning documents that were accepted after resubmission.

## RESULTS

**Figure 1:**
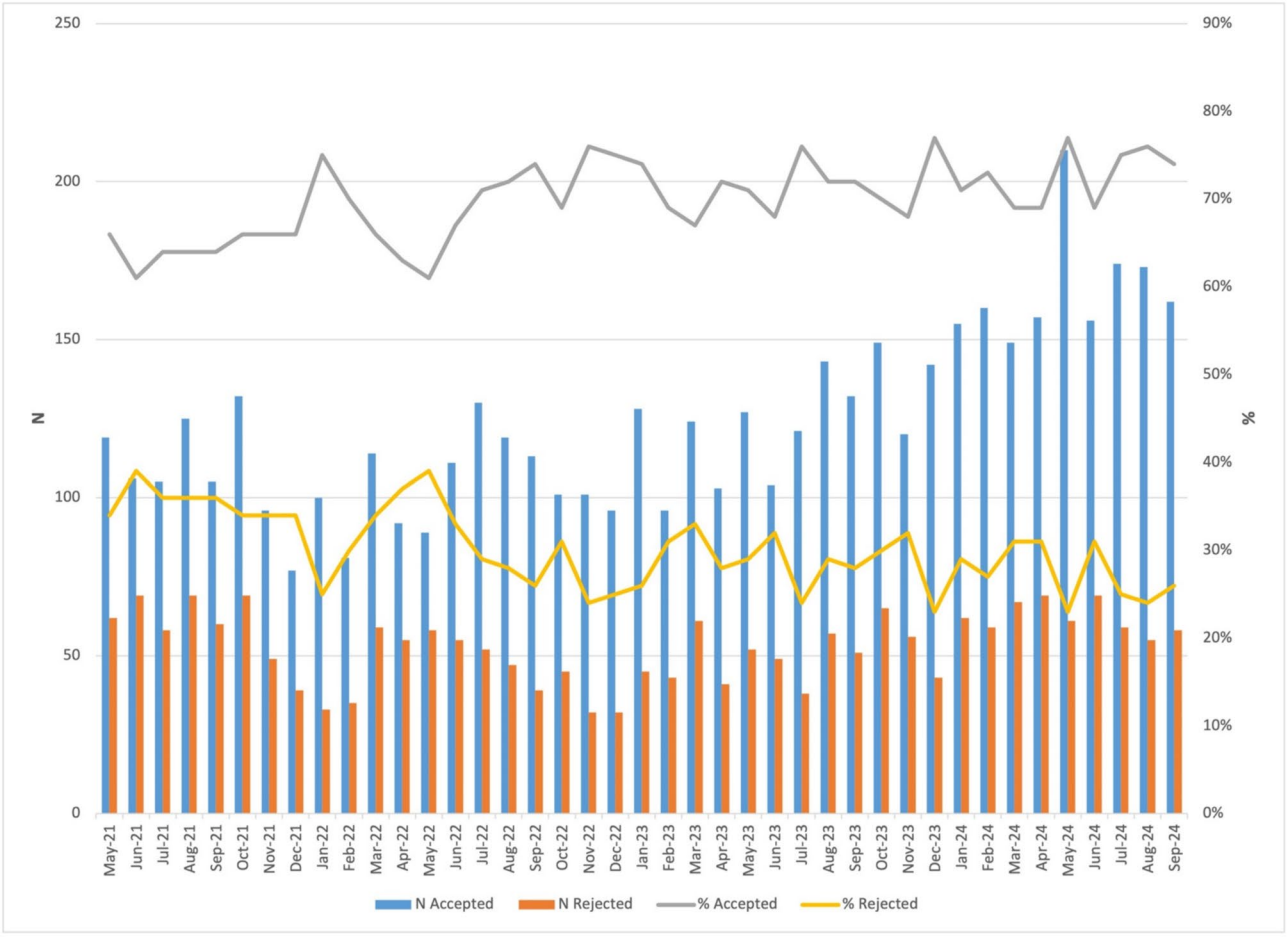
Advance care planning documents accepted and rejected per month

The patient portal began accepting advance care planning documents in May 2021. As seen in Fig. 1, use of the patient portal for uploading documents was immediate with 180 documents uploaded during the first month. Of these, 120 advance directive and POLST forms met the quality standard and were accepted and uploaded into the EHR. Seventy documents were rejected and 8 of these were resubmitted and accepted during the month.

**Figure 2:**
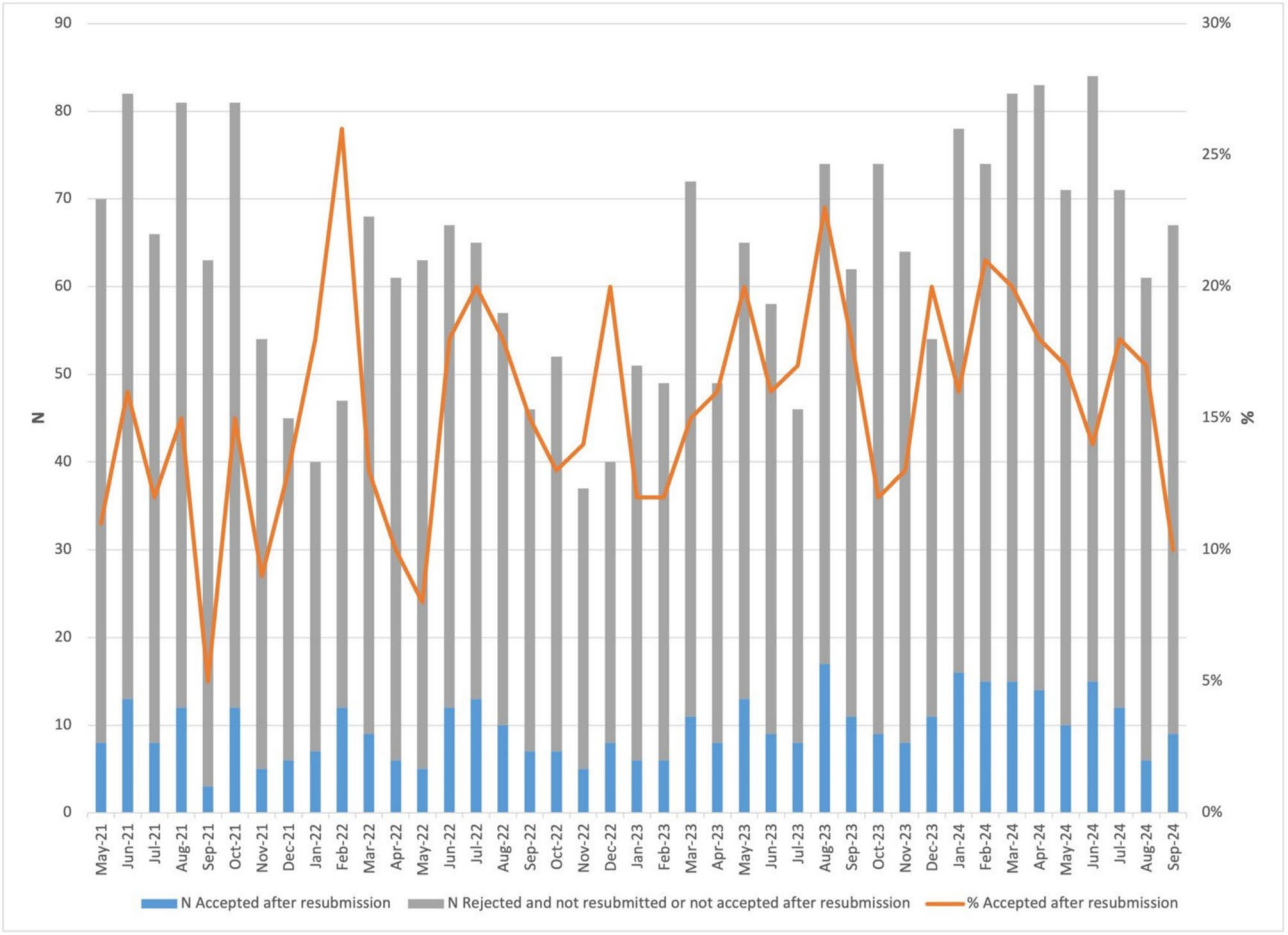
Advance care planning documents rejected and accepted after resubmission, by month

Over the 41-month study period, 7274 advance care planning documents were uploaded via MyChart into the EHR (mean 177 documents per month). During that period, 4700 (65%) documents were accepted on first submission and 2574 (35%) documents were rejected. Of these rejected documents, 397 (15%) were resubmitted and accepted in the same calendar month. While document submission varied by month, there was a gradual increase in the number of submitted advance care planning documents over the studied period (increase of 1.9 documents per month, *p* < 0.0001) and a gradual increase in the proportion of accepted documents (increase of 0.23% accepted per month, *p* < 0.0001). Among rejected advance care planning documents, the proportion that was resubmitted and accepted within the month did not increase in a statistically significant fashion (increase of 0.13 documents accepted after resubmission per month, *p* = 0.09) (Fig. 2). Overall, 5097 (70%) of advance care planning documents were accepted (on submission or re-submission) into the EHR during the study period. This included 4852 (95%) advance directives, 74 (1.5%) POLST forms, and 171 (3.4%) other documents. Advance directives submitted through the patient portal accounted for 19.1% of all the advance directives collected by the health system over the study period. This proportion increased from 14.0% in 2021, 17.3% in 2022, 20.1% in 2023, and 24.4% in 2024.

**Figure 3:**
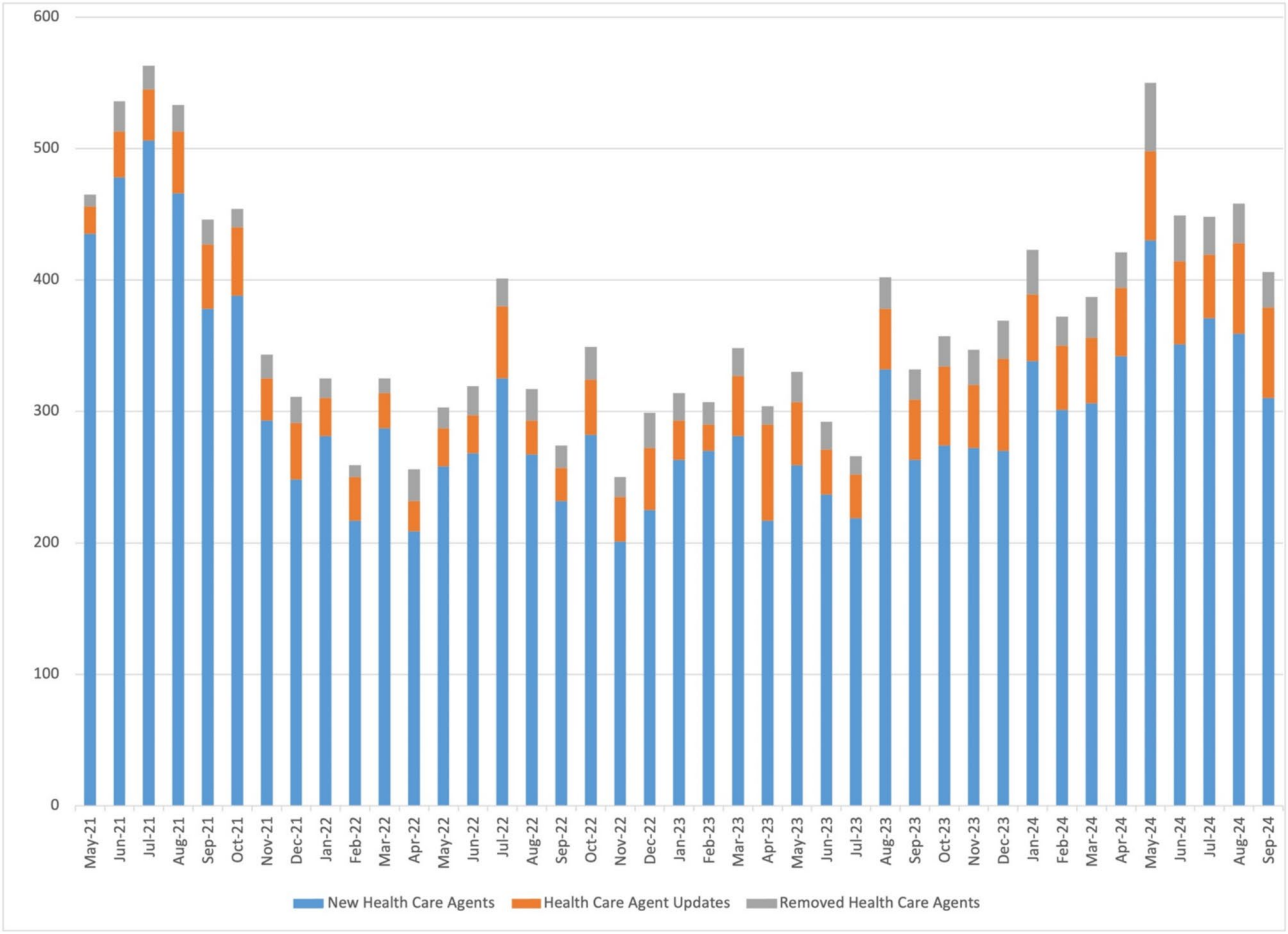
Health care agents added, updated, and removed, by month

During the first month of the patient portal function, 435 new Health Care Agents and Designated Surrogates were entered by patients via MyChart. This included surrogate information entered by the patient and Health Care Agents added to the Advance Care Planning activity by HIIMS because an advance directive was submitted by a patient. Additionally, 21 Health Care Agents (or Designated Surrogates) were updated by patients and 9 Health Care Agents were removed. The frequency of Health Care Agent (and Designated Surrogate) submissions, updates, and removals varied substantially over the 41 months studied. Overall, 12,509 Health Care Agents (or Designated Surrogates) were added over the 41 months (mean 305 per month), 1790 Health Care Agents were updated and 911 were removed (Fig. 3). There was no statistically significant trend in the number of surrogates submitted per month (*p* = 0.99).

## DISCUSSION

Even when patients complete advance care planning instruments, their inclusion within the EHR has proven difficult. This is despite increasing patient access to EHR portals.^8^ We demonstrate that a collaborative mechanism of engaging HIIMS personnel in the collection of these documents can be combined with a common EHR portal to facilitate advance care planning document capture. Furthermore, the structure incorporates a check on quality and feedback to patients for incomplete or incorrect documents. Patient access to information through an EHR portal has been described as a source of empowerment.^9^ This process might facilitate patient engagement in advance care planning.

The number and proportion of accepted documents increased over time, and those submitted through the patient portal became a larger proportion of submitted advance care planning documents over the study period. This likely reflected increasing awareness of primary care physicians concerning the availability of portal submission and increased use of the visit-completion “After visit summary” to direct patients to use the patient portal for advance care planning activities. EHR modifications can be used to address barriers to the administrative aspects of advance care planning,^10^ letting health systems focus on the fundamental obstacles of clinician facilitation of consideration of prognosis, goals, and values.^11^

The capability to easily retrieve and process advance directives is just one step among the many in accomplishing the important task of advance directive completion, but it can fit into interventions to promote advance care planning such as automated patient or physician prompts, navigator outreach, and notaries in clinic. But this mechanism likely raises more questions than it answers, both practical and clinical. For instance, does the clinician become aware of the portal-submitted document? Is it problematic to bypass the doctor-patient interaction that might result in an advance care planning conversation prompted by the document? Do patient-submitted Health Care Agents and Designated Surrogates facilitate decision making in the setting of loss of capacity? Furthermore, we need to learn more about the rejected documents, only a minority of which are accepted after resubmission within the month. We also need to learn about the patients who take advantage of this mechanism. Have these patients communicated with their providers about advance care planning? Does this mechanism reduce or widen well-recognized racial and ethnic disparities?^12^ These questions require additional research. The answers might guide the advancement of interventions such as that presented here and also interventions to improve advance care planning and its clinical implications.

Development of this interdisciplinary approach requires substantial buy-in from health system administration and HIIMS. Resources are needed to train HIIMS personnel and to support their continued participation in the activity. In steady state, the program requires one HIIMS FTE. An advantage of this training process is that HIIMS personnel became more knowledgeable about the clinical area of advance care planning, and they broadened their scope of practice. The training sessions were lively interactive exchanges that permitted these staff to advance their skills from processing of information to participating in the generation of material in the EHR that will directly affect care. This interdisciplinary collaboration facilitates health activities to be distributed in a rational fashion to individuals well-suited to the task, which must be done in a deliberative way,^13^ and can lead to increased efficiency and productivity,^14^ for time-constrained clinicians.^15^

This report aims to provide a blueprint that reduces the effort to develop a patient portal mechanism for submission of advance care planning documents in other health systems. The materials would need to be modified to reflect system workflows and state laws and regulations. The described intervention was implemented in a single academic health system, but the components should be applicable to any system with an EHR that has a patient portal that can accept upload of an advance directive. Furthermore, our report is limited by the fact that we were not able to distinguish between Health Care Agents and Designated Surrogates. We also were unable to retrieve the reasons that documents and surrogates were rejected. Linkage of portal-uploaded advance care planning documents and surrogates to clinical outcomes was beyond the scope of this report.

Advance care planning is a complex process that requires many steps. We describe a multidisciplinary approach to reduce the barrier to getting the completed advance care planning document and surrogate into the EHR with a quality review. This small step is just one of many necessary for advance care planning discussions to guide decisions before and at the point of care.

## Supplementary Information

Below is the link to the electronic supplementary material.

ESM1 (DOCX 1.00 MB)

## Data Availability

No statement is warrated. There are only summary data presented.
