# Supplementary material for: Using the Electronic Health Record Patient Portal to Collect Advance Directives and Surrogate Specification
Source: J Gen Intern Med. 2026 Jan 23;41(9):2395–403. doi: 10.1007/s11606-025-10165-w (PMC13303996; doi:10.1007/s11606-025-10165-w)
Supplement: Supplementary file 1 — (DOCX 1.00 MB) [file 11606_2025_10165_MOESM1_ESM.docx]

**Supplementary Appendix**

**Supplementary Appendix Table. HIIMS Frequently Asked Questions about Portal Uploaded Advance Directives**

| Question | Answer |
| --- | --- |
| How do we process Advance Directive documents that do not include HCAs? | - This is a Living Will. Accept the document as an Advance Directive Enduring. |
| How do we process End of Life DNR documents? | - Accept as an Advance Directive Enduring, if the patient signature and date are present, along with the two witness or notary signature(s) and date(s). |
| How should we process documents entitled Durable Power of Attorney? | - If the document is a Durable Power of Attorney for Health Care or a Power of Attorney for Health Care, process the document as an Advance Directive Enduring. - If the document is a Financial Power of Attorney, deny the document. Reason: Invalid Form. Reason given to patient: Not an Advance Directive document. |
| Should we accept a document that outlines a person is resigning as a Health Care Agent? | - Yes. Accept the document as an Advance Directive Enduring. |
| How do we process records submitted that are not an Advance Directive, POLST, or Living Will? | - For Outside Records: Print/save off the documents and upload via ICAP. Deny the document. Reason: Invalid Form. Reason given to patient: Not an advance directive document. Include a note to explain that the submitted records will be uploaded into the UCLA chart. - For Marriage License: Deny the document. Reason: Invalid Form. Reason given to patient: Not an advance directive document. |
| What should we do when the patient submits an Advance Directive as separate documents for each page? | - Print/save off the separate documents for each page, combine to a single file, and upload via ICAP. Deny the InBasket messages for each individual page. Reason: Invalid Form. |
| Do we deny an Advance Directive that is missing pages? | - Yes. Deny the document. Reason: Missing Documentation. Reason given to patient: Document is Missing Pages. Please re-submit document with all pages. |
| On an Advance Directive, the witnesses’ signatures on the document have no date. Should we accept it? | - Yes. Accept the document as an Advance Directive Enduring. |
| If we have a document that appears to contain all pages, signatures, and dates but it is very blurry and hard to read, what should we do? | - Deny the document. Reason: Illegible. Reason given to patient: Document is illegible or poor quality image. Please re-submit legible document. |
| What should we do when the patient submits a document that includes pages for a different patient? | - Deny the document. Reason: Contact name and document do not match. Reason given to patient: Document associated with an incorrect patient. Include a note to explain that the patient submitted documents for different people together, and they should re-submit the documents separately. |
| A patient submitted a POLST. The patient also submitted the names of individuals to include as Health Care Agents. Should we enter the individuals as Designated Surrogates? | - Yes. If there is no Advance Directive to support the individuals as Health Care Agents, they are Designated Surrogates. |
| In a POLST document, page 1 of 2 is complete. Page 2 is blank. Should we accept it? | - Yes. The second page is not required. |
| What should we do when the patient submits an Advance Directive with fillable fields, and the form is editable? | - First, make the document non-editable. (See outlined steps below). Upload the non-editable version to ICAP. Deny the InBasket messages for the fillable document. Reason: Invalid Form. Reason given to patient: Include a note to explain that the submitted document was editable. Therefore, a non-editable version has been uploaded into the UCLA chart. |

**Supplementary Appendix Figure 1. Modified Epic Advance Care Planning activity to facilitate advance care planning**


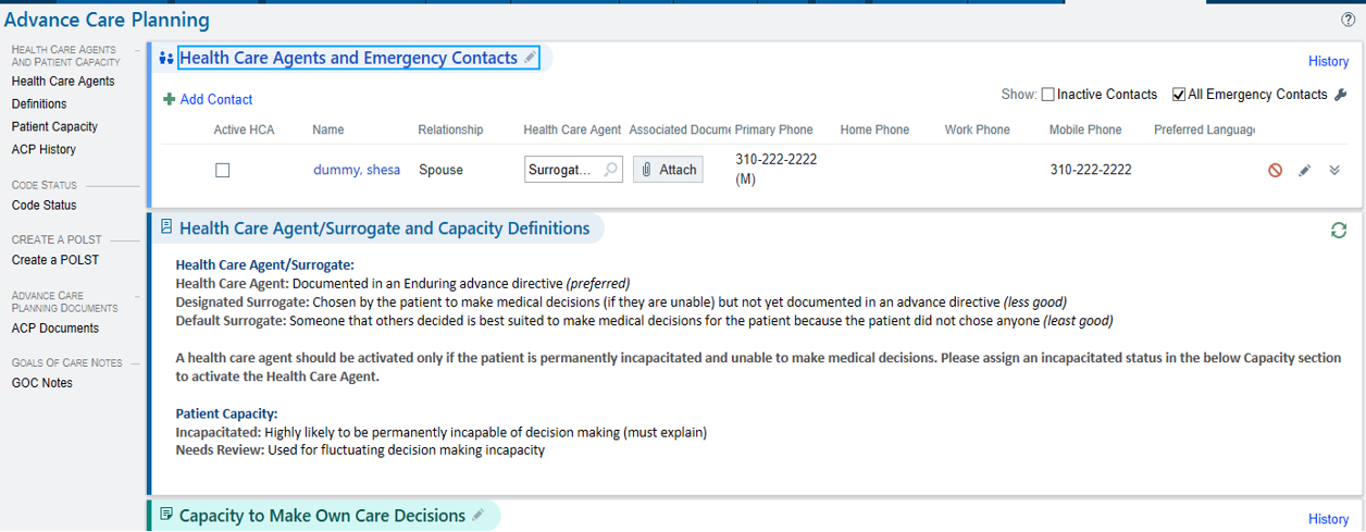


**Supplementary Appendix Figure 2. Modified MyChart Advance Directive page including document and surrogate upload capability.**

**
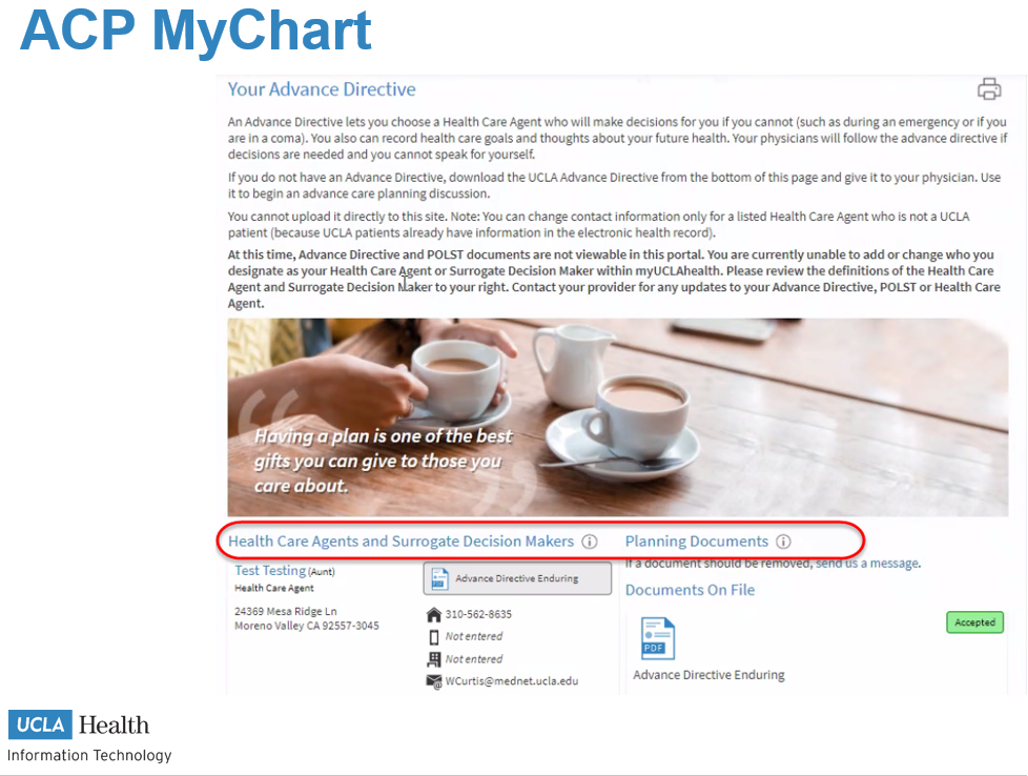
**
